# How Do Ecosystem Services Affect Poverty Reduction Efficiency? A Panel Data Analysis of State Poverty Counties in China

**DOI:** 10.3390/ijerph19031886

**Published:** 2022-02-08

**Authors:** Peng Cao, Xiao Ouyang, Jun Xu

**Affiliations:** 1 School of Mathematics and Statistics, Hunan Normal University, Changsha 410081, China; cp@hunnu.edu.cn; 2 Hunan Institute of Economic Geography, Hunan University of Finance and Economics, Changsha 410205, China; xujun19900531@gmail.com

**Keywords:** state poverty counties, poverty reduction efficiency, ecosystem services, mechanism framework, panel analysis

## Abstract

Scientific evaluation of the interaction between poverty reduction efficiency (PRE) and ecosystem services (ES) in state poverty counties is essential in promoting the rural revitalization strategy and the construction of an ecological civilization. Using the DEA model, the InVEST model, and fixed-effect panel data, this study was analyzed using the panel data of 832 poverty counties in China for 2010–2019 to evaluate the relationship between poverty reduction efficiency and ecosystem services. The main results are as follows: (1) The overall poverty reduction efficiency showed an upward trend, while ES exhibited a declining trend with spatial heterogeneity. The poverty reduction efficiency of state poverty counties in the western region increased rapidly. (2) The impact of different types of ecosystem services on poverty reduction efficiency varied considerably. Habitat quality was significantly negatively impacted, while food production and carbon storage showed significant positive effects. There was a significant positive relationship between ecosystem services and poverty reduction efficiency in all regions, with the eastern region having the strongest correlation. (3) The panel regression analysis showed a significant positive impact. The environmental parameters were the primary factors affecting poverty reduction efficiency, while economic and social factors were the driving and external factors. The rural revitalization strategy should strive towards the win-win effect of ecological protection and economic development.

## 1. Introduction

Poverty does not only affect the economy and people’s livelihoods, but also the social stability and long-term development of a country. Countries across the globe have pledged to eradicate poverty as part of their commitment to achieving the UN Sustainable Development Goals (SDGs). China, the most populous country in the world, used to face severe poverty and other socio-economic issues in rural development [1]. From 2013 to 2020, China adopted “The Targeted Poverty Reduction Policy” to eradicate extreme poverty in rural China, lifting nearly 100 million poor people out of poverty. This resulted in the removal of all counties in China from the poverty list and resolved major regional poverty problems, thereby supporting international efforts to curb global poverty [2].

Ecosystem services have always been the focus of research. Since most of China’s poor areas overlap with ecologically fragile areas and key ecological function areas, poverty reduction must balance and consider environmental protection to achieve green sustainable development [3]. Many decision makers and scholars have focused on policies and strategies promoting both ecosystem services and poverty reduction efficiency, realigning poverty reduction measures based on the ecological environment and resource endowments in marginalized communities. In this paper, we used the fixed-effect panel model to explore the non-linear relationship between ecosystem services and poverty reduction efficiency. This study can help support the dualistic ecological protection and poverty reduction strategy and provide a reference for implementing “The Targeted Poverty Reduction Policy” and “The Rural Revitalization Strategy”.

## 2. Literature Review

Poverty reduction efficiency refers to the maximum possible output that the government can achieve in poor areas through investments in human, material, and financial resources and other poverty reduction elements under established technical conditions. It is an important indicator measuring the efficiency of poverty-reduction element allocation [4]. Ecosystem services support human well-being and are essential in the integration of environmental needs in national policy [5]. Ecosystem services help improve poverty reduction efficiency in poverty-stricken areas and are crucial to maintaining family livelihoods [6].

Studies on poverty reduction efficiency and ecosystem services have tackled the definitions, evaluations, and mechanisms of poverty. Definitions of poverty and indicators gauging poverty have varied from scholar to scholar. One such indicator is household income or consumption poverty, which determines poverty based on a given threshold in terms of income or consumption [7]. Alkire and Foster (2011) proposed a multidimensional poverty theory [8], which suggests that poverty deprives families of their rights in various aspects of welfare (e.g., income, education, medical care). In 2002 the World Bank put forward the theory of vulnerability to poverty that measures the level of risk of falling into poverty in the future [9].

Previous studies have also used poverty reduction efficiency in assessing different poverty reduction projects from various dimensions. For instance, some studies developed a poverty reduction quality index in political, economic, and cultural dimensions to evaluate poverty reduction [10], while others have used a quality index for family life, development, and public service [11]. Some have evaluated poverty reduction efficiency for industrial poverty reduction [12], financial poverty reduction [13], tourism poverty reduction [14], and other projects. Different measurement approaches have been used to study the influencing factors of poverty reduction efficiency. Models evaluating poverty reduction efficiency parameters include the stochastic frontier approach [15], regression analysis [16], the TOPSIS method [17], and intuitionistic fuzzy analysis [18]. The influencing factors of poverty reduction efficiency were analyzed based on natural resource endowment [19], socio-economic development [20], and the implementation of poverty reduction policies.

Numerous studies have also focused on the effects of ecosystem services on poverty reduction efficiency. Some have analyzed the impact of ecosystem services on household income poverty or multidimensional poverty, the interaction between ecosystem services and livelihood activities in poor communities [21], and the poverty reduction effects of different ecosystems (e.g., farmland ecosystems, forest ecosystems) [22]. While considerable research has been conducted on the efficiency of poverty reduction and ecosystem services, more studies are needed in three aspects: (1) While scholars have constructed multidimensional poverty indices or quality indices of poverty reduction from different dimensions to measure the effect of poverty reduction, nearly all of them measured poverty reduction efficiency in terms of the output of poverty reduction resource elements. Few combined input and output to evaluate poverty reduction efficiency. (2) Most studies used cross-sectional data from a particular province or impoverished community to analyze the relationship between ecosystem services and poverty reduction efficiency. Since most do not use long-term panel data, the evolutionary characteristics of poverty are difficult to identify. (3) Previous studies have mainly focused on research on the impact of ecosystem services on household livelihood status or regional economic development. Few have investigated the interaction between ecosystem services and poverty reduction efficiency, and even fewer have analyzed its impact mechanisms.

China has made considerable efforts to reduce poverty. However, with the declining marginal effects of poverty reduction, some impoverished areas with fragile ecological environments and less developed economies face higher risks of returning to poverty [23]. Understanding the interaction between ecosystem services and poverty reduction efficiency is crucial for rural revitalization and the construction of an ecological civilization. Based on this premise, this study aims to answer the following research questions: (1) Is there a connection between the poverty reduction efficiency of impoverished counties and ecosystem services, and what is the extent of the impact? (2) Are there differences in the impacts of different types of ecosystem services on poverty reduction efficiency? (3) Are there significant differences in the impacts of ecosystem services at different regions on poverty reduction efficiency? The results of this study can help provide a win-win situation for economic development and ecological protection, offering an effective and scientific theoretical basis on the coupling of poverty reduction and sustainable rural revitalization.

## 3. Study Area, Analytical Methods, and Data Sources

### 3.1. Study Area

**Figure 1:**
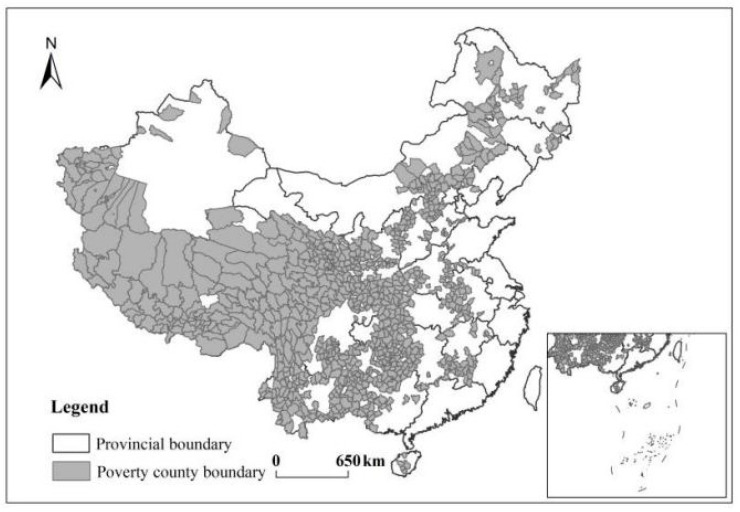
Study area.

We selected state poverty counties (to be referred to as poverty counties) in China as the research object. A total of 832 national key counties for poverty reduction and development announced by the Leading Group Office of Poverty Reduction and Development of the State Council in 2014 were used in the study (http://www.cpad.gov.cn, accessed on 14 October 2021), as seen in Figure 1. The poverty counties are distributed in 22 provinces, autonomous regions, and municipalities, mainly in areas with fragile ecological environments and slow economic development. They are distributed in the central and western regions. The counties are located at 18°23′–51°25′ N and 73°40′–135°2′ E, with average altitudes of 2.90 km. The study area has 11,277.329 m^2^ in construction land (2019), a per capita income of less than 1300 yuan (old revolutionary areas and minority border areas less than 1500 yuan), and a poverty population of 70.17 million (2014).

### 3.2. Analytical Methods

#### 3.2.1. Evaluation Model of Poverty Reduction Efficiency

1. Data envelopment analysis model

Previous studies have generally measured efficiency using the stochastic frontier approach (SFA) and data envelopment analysis (DEA) methods. The DEA method has the following advantages: First, the DEA method does not need to set a specific function form and can deal with multiple comparable indicators, thereby avoiding the structural deviation caused by the incorrect setting of the production function in other calculations (e.g., SFA) [15]. Second, the calculated efficiency value can be divided into the technical progress index and the technical efficiency index. This is especially useful in the ecosystem services evaluation of multiple inputs and outputs [24].

Since development-orientated poverty reduction mainly relies on investments in human, material, and financial resources to help poor communities alleviate poverty, we chose the DEA model to evaluate the poverty reduction efficiency of poverty counties using multiple inputs and outputs. The calculation formula is as follows:

(1) $$min\left[\theta -\varepsilon \left(\sum_{i=1}^{m}si^{-}+\sum_{r=1}^{n}sr^{+}\right)\right].\;s.t.\{\begin{array}{c} \sum_{j=1}^{I}x_{ij}\lambda_{j}+si^{-}=\theta x_{ik}\\ \sum_{j=1}^{I}y_{rj}\lambda j+si^{+}=y_{rk}\\ \sum_{j=1}^{I}\lambda_{j}=1,j=1,2,\ldots ,n \end{array}$$

where $\lambda_{j}$, $si^{-}$, and $si^{+}\geq$ 0. We assume that the BCC model has multiple decision-making units (DMU), such that $x_{ij}\geq 0$ is the *i*-th input index of a certain decision-making unit *j.* $y_{rj}\geq 0$ is the output of the *r*-th item of a certain decision-making unit *j*, θ is the target planning value, $\lambda_{j}$ is the planning decision variable, ε is the non-Archimedean infinitesimal quantity, and $si^{-}\;\mathrm{and}\;si^{+}$ are slack variable vectors. If θ=1  and $s^{-}=0$ or $s^{+}=0$, the DMU is DEA-efficient. If θ<1, the DMU is DEA-non-efficient. If θ=1 and $s^{-}$ ≠ 0 or $s^{+}$ ≠ 0, the DMU has a weak DEA efficiency.

In order to reflect the rationality of the assumption, the variable returns to the scale of the $BC^{2}$ model that was used. The comprehensive efficiency calculated by DEA was decomposed into technical efficiency and scale efficiency for analysis, such that comprehensive efficiency is equal to the product of technical efficiency and scale efficiency [25].

2. Selection of input and output indicators

Based on the existing poverty reduction evaluation index system and considering data accessibility issues [4], we constructed the following DEA model evaluation index system: the input indicators include county financial fund investment, the amount of employment in the secondary and tertiary industries, construction land, and internal expenditures for research and experimental development. Output indicators include rural annual per capita net income and non-poverty incidence. The specific input indicators and output indicators were shown in Table 1. The above indicators related to prices were deflated based on 2010 to make the results comparable. 

#### 3.2.2. Evaluation Model of Ecosystem Service

Based on the recommendations of related studies [26], we characterized ES supply using three indicators: habitat quality (HQ), food production (FP), and carbon storage (CS). The range method was used to eliminate the unit differences in supply for different ecosystem services, and the average was calculated and used as the value of ES.

1. HQ

HQ refers to the ability of the ecological environment to provide suitable conditions for individuals or groups. Using InVEST’s HQ module, the land cover data was combined with the biodiversity threat factors to calculate the raster data [27]. The formula is as follows:

(2) $$zQ_{xj}=H_{j}\left[1-\left(\frac{D_{xj}^{z}}{D_{xj}^{z}+k^{z}}\right)\right]$$

where $Q_{xj}\;$ is the habitat quality of the grid *x* in the habitat type *j*, $D_{xj}\;$ is the disturbance degree of the grid *x* in the habitat type *j*, *k* is the half-saturation constant, which is usually half of the maximum value obtained after a trial run of $Q_{xj}$, and $H_{j}$ is the habitat suitability of habitat type *j*.

2. FP

FP is an important indicator in the evaluation of the ecosystem service supply, especially in agricultural ecosystems, serving a vital role in human survival and development. Different land-use types produce different foods. We used the total food output value per unit area of different land-use types to characterize food supply capacity [28] using the formula:

(3) $$G_{i}=\sum G_{ij}=\sum \frac{L_{ij}}{S_{ij}}$$

where *i* represents different administrative regions, *j* refers to the different land-use types, *G* is the total food output value per unit area for the different land-use types in different administrative regions, *L* is the total food output value for the different land-use types in different administrative regions, and *S* is the area of different land-use types in different administrative regions.

3. CS

CS is an important regulating ecosystem service, which has a crucial role in maintaining the above-ground carbon balance of terrestrial ecosystems. The CS module in the InVEST model was used to assess the supply of CS services in poverty counties [29]. The CS module of the InVEST model contains four basic carbon pools: above-ground biological carbon, below-ground biological carbon, soil carbon, and carbon stocks in dead organic matter. The average carbon density of the carbon pools for the different land types was calculated using the land-use data. The area of each land-use type was then multiplied by its carbon density and summed up to obtain the total CS in the study area. The calculation formula is as follows:

(4) $$C_{tot}=C_{above}+C_{below}+C_{soil}+C_{dead}$$

where $C_{tot}$ is the total CS of the urban agglomeration (unit: t·hm^−2^), $C_{above}$ is the above-ground biological carbon (unit: t·hm^−2^), $C_{below}$ is the below-ground biological carbon (unit: t·hm^−2^), $C_{soil}$ is the soil organic CS (unit: t·hm^−2^), and $C_{dead}$ is the carbon stocks in dead organic matter (unit: t·hm^−2^).

#### 3.2.3. Analysis Model of the Impact of PRE on ES

While the DEA model can objectively evaluate the relative efficiency of decision-making units and calculate the poverty reduction efficiency of state poverty counties, it is unable to analyze the mechanism of natural, economic, and social factors affecting poverty. For this study, we selected the fixed-effect panel model to analyze the impact of the value of ecological service on the efficiency of poverty reduction [30].

The dependent variable was poverty reduction efficiency (PRE), which was decomposed into two dimensions: technical efficiency (TE) and scale efficiency (SE). The main explanatory variable was the ES, which was split into three dimensions: HQ, FP, and CS. Control variables included the natural environment, economic development, and social factors. Precipitation (preci), temperature (temp), and vegetation index (ndvi) were used to characterize the natural environment [31], agricultural output value (agri), and tourism income (tourism) for economic development [32], and education expenditure (edu) and financial loans (debt) were used for the social factors [33]. The model is described as follows:

(5) $$PRE_{it}=\beta_{0}+\beta_{1}ES_{it}+\beta_{2}control_{it}+year_{t}+\varepsilon_{it}$$

where $PRE_{it}$ refers to the comprehensive poverty reduction efficiency of the *i*-th poverty county in year *t* (*i* = 1, ⋯, 832; *t* = 2010, ⋯, 2019), $ES_{it}$ is the ecosystem service, $control_{it}$ includes the different control variables, $year_{t}$ is the time effect, and $\varepsilon_{it}$ is the random disturbance item that conforms to the gaussian distribution. The parameters to be estimated are $\beta_{0},\;\beta_{1},\;\mathrm{and}\;\beta_{2}$, among which $\beta_{1}\;$ is used to measure the effects of ecological services on poverty reduction efficiency. The description of the variables is shown in Table 2.

### 3.3. Data Source

Data for the various indicators, i.e., financial fund investment, employment, research and development expenditure, net income per capita, agricultural output value, tourism income, education expenditure, and financial loans, were from 2010 to 2019 and were derived mainly from provincial statistical yearbooks. The construction land data were obtained from the Resource and Environment Science and Data Center of the Chinese Academy of Sciences (http://www.resdc.cn/, accessed on 12 October 2021), while the datasets for poverty incidence were from the Poverty Monitoring Report of Rural China and the National Bureau of Statistics.

HQ comprised the following parameters: habitat suitability, stress factor weights, maximum stress distance, sensitivity of habitat type to stress factors, and other related parameter settings for each habitat category (e.g., carbon density under different land-use types). For the HQ parameter selection, we referred to the research results [26]. The FS data were derived from the statistical yearbooks of counties, cities, and districts. Total agricultural output value (agri) corresponds to the area of arable land, the total forestry output value to the area of woodland, the total output value of animal husbandry to the area of grassland, and the total value of fishery to the water area. Data for precipitation (preci), temperature (temp), and vegetation index (ndvi) were obtained from the Resource and Environment Science and Data Center of the Chinese Academy of Sciences (http://www.resdc.cn/, accessed on 10 October 2021), with a 1 km × 1 km spatial resolution and WGS1984 UTM Zone 49 N projection coordinate system.

The raw data were first preprocessed. County samples with missing indicators and outliers were removed from the dataset, resulting in 829 as the final research object count. Indicators with currency units were log-transformed to eliminate the influence of heteroscedasticity. Finally, to avoid “false regression”, the unit root test was applied to verify the stationarity of each variable. The unit root test results show (see Appendix A Table A1) that the variables were stable and could be directly estimated using the panel model. The descriptive statistics and correlation analysis of variables are shown in Appendix A Table A2 and Table A3.

## 4. Empirical Results and Analysis

### 4.1. Analysis of PRE in Poverty Counties Based on DEA Method

**Figure 2:**
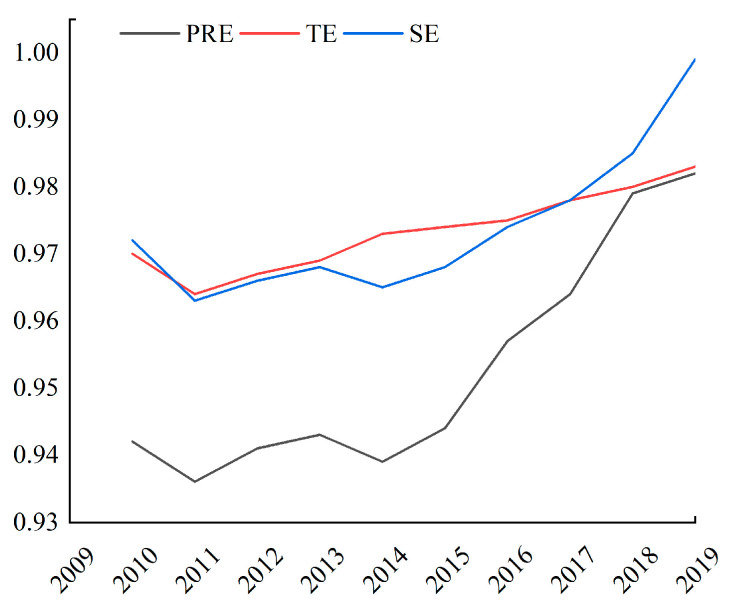
Change trend of the subdimensions of PRE for 2010–2019.

The poverty reduction efficiency (PRE) showed a fluctuating upward trend for the given study period in Figure 2. The time–series changes presented phased characteristics in general. The comprehensive poverty reduction efficiency in poverty-stricken counties gradually increased from 2010–2013, decreased slightly in 2013–2014, and exhibited an upward trend in 2014–2019 with a 4.5% increase.

**Figure 3:**
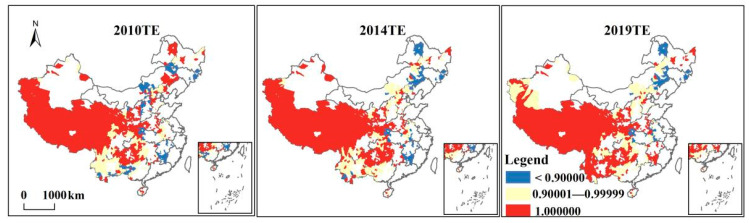
Changing trends in technical efficiency (TE) of national poverty counties, 2010–2019.

Based on the recommendations of previous studies [25], poverty reduction efficiency was decomposed into technical efficiency (TE) and scale efficiency (SE), and their changing trends were observed. The technical efficiency of poverty reduction showed a slight increase in fluctuation (Figure 1). The technical efficiency of the southwestern region displayed a significant growth trend during the study period. This could have been caused by vigorous developments in the digital economy, intelligent manufacturing, electronic information, and other emerging industries in Sichuan and Chongqing, providing strong support for improving poverty reduction efficiency in poor areas (Figure 3). Likewise, governments at various levels have increased poverty reduction efforts in many contiguous and extremely poor areas. Local governments have adopted relatively effective fund management methods and have improved their capital allocation and utilization capabilities. These measures resulted in a rebound in the value of pure technical efficiency in the region, exerting corresponding output benefits and steadily increasing technical efficiency.

The scale efficiency showed an overall upward trend for the given research period (Figure 1). From 2010 to 2016, the scale efficiency did not change considerably. From 2016 to 2019, the scale efficiency had a pronounced increasing trend, reaching 1 in 2019. The results suggest that with the continued increase in financial investments into poor counties, the industrial structure of the county economy can be optimized, and the positive effects brought by scale efficiency can be exerted.

### 4.2. Spatio-Temporal Analysis of ES in Poverty Counties

**Figure 4:**
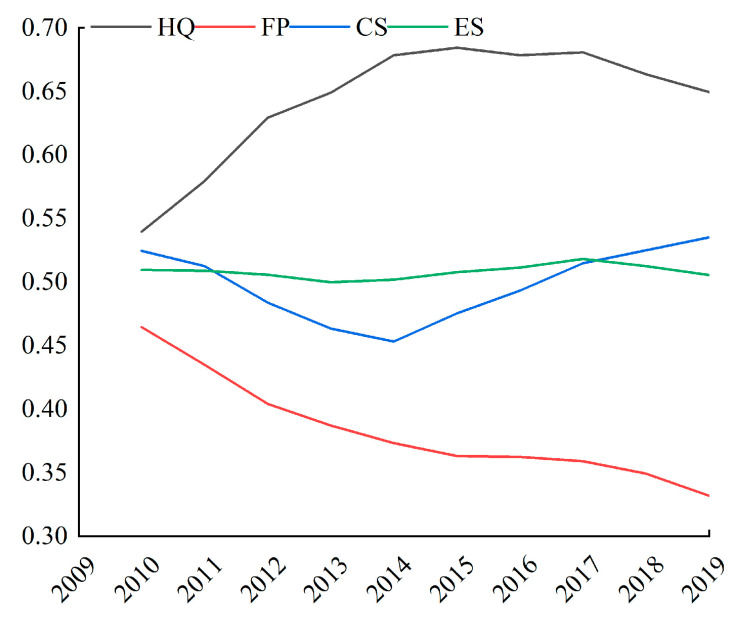
Change trend of subdimensions of ES in 2010–2019.

As shown in Figure 4, ES showed a slight fluctuation trend throughout the survey years. In 2010, 2014, and 2019, the ES value was 0.5095, 0.5018, and 0.5054, respectively, resulting in a downward trend. From the indicators of different dimensions, HQ generally increased first and then remained stable, with an average annual increase of 0.0242 units from 2010 to 2015, and then remained stable after 2015. FP showed a decreasing trend throughout the survey period. The change of CS shows a trend of decreasing first and then increasing, reaching the minimum in 2014, and then showing an upward trend.

**Figure 5:**
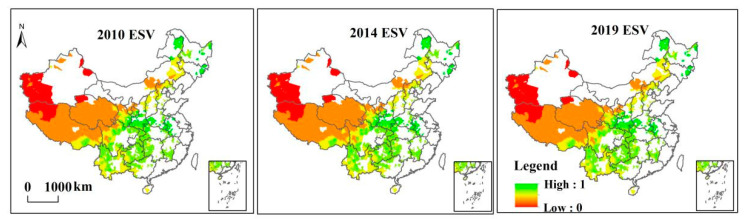
Spatial distribution of ES in national poverty counties.

In Figure 5, the change in ES has been decreasing spatially. Low ES areas were distributed in the northwest (including Gansu, Qinghai, Ningxia, and Tibet), mainly due to natural conditions such as high altitude, dry climate, and low vegetation coverage. Other low ES areas were found in the North China Plain (including Beijing, Tianjin, southern Hebei, Shandong, Jiangsu, Anhui, and eastern Henan), the Sichuan Basin, and the Northeast Plain (including northeastern Inner Mongolia). The low ES intensity could have been caused by the enormous pressure from social and economic development and urbanization in these regions. This means that the development of relevant industries in these poor areas may affect the local ecological environment, resulting in a decreasing trend in ES.

### 4.3. Spatio-Temporal Analysis of ES in Poverty Counties

#### 4.3.1. Benchmark Regression Results

STATA 16.0 was utilized for panel data analysis. The Hausman test was conducted to determine whether to use the panel fixed-effects or random-effects model. The corresponding chi-square value of the Hausman test was 325.72, and the adjoint probability was much less than 0.05. Therefore, the fixed-effects panel model analysis was used. The summary of the results is shown in Table 3.

Ecosystem services were found to have a significant positive impact on poverty reduction efficiency. As shown in Columns (1), (3), and (5) of Table 3, ecosystem services had a positive effect on poverty reduction efficiency, technical efficiency (TE), and scale efficiency (SE). Columns (2), (4), and (6) show the regression results after introducing the control variables (i.e., regional economy, society, and natural conditions). Based on the results, the conclusion did not change after the control variables were introduced. Specifically, the results in columns (1) and (2) show that after introducing the control variables, PRE increased by 0.274 units on average for every unit increase in the value of ecosystem services. In Columns (3) and (4), the value of ecosystem services had a strong positive impact on the TE for state poor counties before and after the introduction of control variables. Columns (5) and (6) show that before and after the inclusion of control variables, ES value had a positive influence on scale efficiency.

Tourism income (tourism) and education expenditure (edu) had significant positive effects on poverty reduction efficiency, indicating that these two parameters are important in improving the poverty reduction efficiency of state poverty counties. Agricultural output value (agri) and financial loan (debt) were found to have significant negative relationships with the poverty reduction efficiency of state poverty counties. The vegetation index (ndvi) and regional temperature (temp) had positive correlations with poverty reduction efficiency, but they were not statistically significant. Rainfall had a significant negative effect, which means that increased rainfall could increase the risk of economic losses due to natural disasters and reduce poverty reduction efficiency.

#### 4.3.2. Robustness Analysis

The robustness test was carried out by changing the dummy variable into a dependent variable. If the poverty reduction efficiency (PRE), technical efficiency (TE), and scale efficiency (SE) are greater than the average value for each year, the variable was defined as 1; otherwise, the value would be 0. Then, the logit model was used for empirical analysis. As shown in Table 4, the change of the dummy variable into a dependent variable would not change the conclusions of this paper.

#### 4.3.3. Heterogeneity Analysis

1. Different dimensions of ES

The effects of particular sub-dimensions of ecosystem services on poverty reduction efficiency were further explored. Columns (1), (2), and (3) of Table 5 show the results when analyzing the impact of habitat quality (HQ), carbon storage (CS), and food production (FP) on poverty reduction efficiency. The results indicate that HQ has a significant negative impact on poverty reduction efficiency, while FP and CS had significant positive effects.

2. Different regions

The impact of ecosystem services on poverty reduction efficiency can be considerably influenced by the region’s economic development level [3]. To analyze the impact of ecosystem service on poverty reduction efficiency in areas with varying economic levels, the study area was divided into eastern, central, and western regions. The division for the eastern, central, and western regions is based on China’s 1986 Seventh Five-Year Plan. The eastern region includes Beijing, Tianjin, Hebei, Liaoning, Shanghai, Jiangsu, Zhejiang, Fujian, Shandong, Guangdong, and Hainan Province, for a total of 11 provinces (or cities). The central region includes Shanxi Province, Inner Mongolia Autonomous Region, Jilin Province, Heilongjiang Province, Anhui Province, Jiangxi Province, Henan Province, Hubei Province, Hunan Province, and Guangxi Province, for a total of ten provinces (or regions). The western region includes Chongqing City, Sichuan Province, Guizhou Province, Yunnan Province, Tibet Autonomous Region, Shaanxi Province, Gansu Province, Qinghai Province, Ningxia Autonomous Region, and Xinjiang Autonomous Region, for a total of ten provinces (cities or regions). Columns (1), (2), and (3) of Table 6 show the effects of ES values for the eastern, central, and western regions on the efficiency of regional poverty reduction. The results show a significant positive relationship between ecosystem service values and poverty reduction efficiency in all three regions. The efficiency in the eastern region was higher than in the central and western regions (0.4527 > 0.3635 > 0.2342).

## 5. Discussion

### 5.1. Analysis of the Mechanism of PRE Based on ES

**Figure 6:**
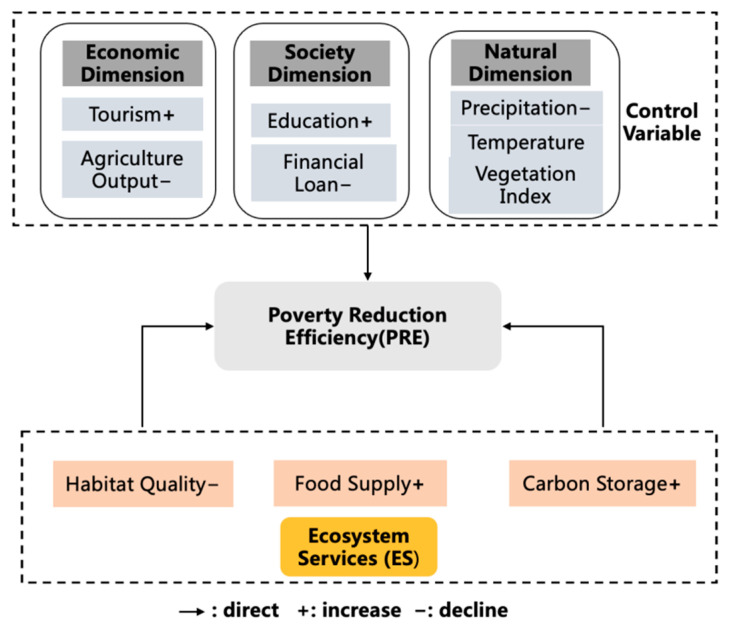
The impact mechanism of PRE on ES.

Figure 6 shows the mechanisms linking ecosystem services (ES) and poverty reduction efficiency (PRE). Research has, to date, focused largely on poverty dimensions concerning income, assets, food security, and nutrition. Few studies have provided sufficient context to enable a thorough understanding of the positive or negative factors affecting poverty reduction efficiency [34]. This paper examines how ecological (i.e., habitat quality (HQ), carbon storage (CS), and food production (FP)), economic (i.e., regional agricultural output value and tourism income), and social factors (i.e., education expenditures and financial loans) impact poverty reduction efficiency. The results suggest that environmental factors are the primary elements affecting poverty reduction efficiency in China’s poverty counties. A healthy and vibrant ecology is fundamental to sustaining biodiversity and maintaining essential ecological functions in the surrounding environment. The vast majority of poor people in China live in rural areas, and their production and living standards are highly dependent on the ecological environment [35].

Economic factors are the driving forces affecting poverty reduction efficiency in state poverty counties. On the one hand, cash subsidies are effective in providing economic compensation for poor households, while releasing household labor for part-time urban employment [36]. On the other hand, tourism income has a significant positive impact on poverty reduction efficiency. The development of tourism broadens the economic income channels, which generates poverty reduction effects [14]. In contrast, agricultural output value has a significant negative effect on poverty reduction efficiency. Families carry out agricultural activities in ecologically fragile areas, such as barren lands, but the low return rate of agricultural labor and exposure to more environmental hazards greatly reduces the efficiency of poverty reduction [37].

Social factors are the external mechanisms that influence poverty reduction efficiency in state poverty counties. Educational expenditure has a significant positive impact on poverty reduction efficiency, increasing human capital so that family members can participate in the workforce, generate regular income, and lift themselves out of poverty [38]. On the other hand, financial loans have a negative impact on poverty reduction efficiency. There are several explanations for this. For instance, Elite Capture and Matthew’s Effect on financial development increase the income gap between groups. Nizam et al. (2020) pointed out that the availability of financial services is largely affected by the income threshold. Given that the degree of inclusion is not high [39], it is often difficult for low-income people to enjoy the development dividend of inclusive finance [40]. Financial loans offered by financial institutions in indigent communities are mostly for industrial purposes and are affected by market changes, operating conditions, and long industrial chains. The effect of poverty reduction is probably not as pronounced in the short term, consistent with the conclusions of previous studies [12].

### 5.2. Analysis of the Mechanism of PRE Based on ES

The findings suggest that ecosystem services (ES) have a positive impact on the poverty reduction efficiency (PRE) of poor poverty counties. While formulating policies promoting rural revitalization and constructing an ecological civilization, governments should harmonize ecological protection and economic growth in day-to-day decision-making and long-term plans towards green sustainable development. Therefore, the following policy recommendations are put forward:

(1) Technical efficiency (TE) and scale efficiency (SE) should be continuously improved, particularly in state poverty counties. Since most of China’s western region is remote with limited development and backward management technology, the central and eastern regions should increase their radiating and leading roles and introduce more capital, technology, and talents to the countryside, and promote balanced and coordinated development. More developed regions should also accelerate the formation of market scale effects when implementing the unified purchase and sale of corresponding characteristic products. By improving production technology and optimizing the allocation of poverty reduction resources, goods and services will be more competitive, generating considerable market scale benefits and promoting sustainable development.

(2) Government should strive towards the win-win effect of ecological protection and economic development. Ecosystem services can effectively accelerate improvements in poverty reduction efficiency, and the ecological environment should not be sacrificed in exchange for economic development. Fragile natural environments should be closely watched, and their ecological functions should be monitored to avoid undermining poverty reduction results. Impoverished areas, particularly in the central region with high ecosystem services, should systematically analyze factors such as resource endowment, market space, and industrial coverage and promote characteristic tourism, ecological agriculture, and other industrial projects that would efficiently increase regional income. Less economically developed counties, particularly in the western region with low ecosystem services value, should strengthen the restoration of the natural environment. These communities should conduct vocational training for rural laborers to increase their work skills and ensure stable employment.

(3) The state should accelerate the completion of needed infrastructure and public service facilities. Through government intervention and financial support, financial support for education can be increased, especially basic education and vocational education. The coverage and strength of social security should also be improved. A more inclusive financial system should be promoted to increase the financial services offered to poor communities and provide financial guarantees to increase poverty reduction efficiency.

### 5.3. Research Limitations and Research Prospects

This paper used qualitative and quantitative analysis methods to measure the poverty reduction efficiency of state poverty counties in China. The results help to identify the temporal and spatial differentiation characteristics of poverty reduction efficiency from a macro-level at the county level in China and provide a scientific reference for the integration of targeted poverty reduction and rural revitalization strategies. However, this study still has certain limitations. For instance, ecosystem services only included habitat quality, food supply, and carbon storage. Further, the cultural–ecological value was not included in the analysis, which may have a considerable effect. Some areas promote economic development through the development of eco-cultural tourism, thereby improving the efficiency of poverty reduction in state poverty areas [41]. Future studies can combine ecosystem services with cultural factors to analyze the effect on regional poverty reduction. Subsequent research may also consider accounting for regional differences in poverty reduction efficiency and the spatial heterogeneity of the impact of ecosystem services. In addition, this study selected only four input and two output indicators to evaluate the efficiency of poverty reduction. Poverty reduction efficiency is closely related to implementing policies in industry and finance, but policy data is difficult to quantify. Future research can explore new parameters according to the impact of different policies and strategies.

## 6. Conclusions

Using the balanced panel data of 832 poverty counties in China for 2010–2019, this study adopted the DEA model to measure the poverty reduction efficiency of impoverished counties and constructed an ES system index. The fixed-effect model was used to analyze the natural, economic, and social factors that affect poverty reduction efficiency, and the following conclusions have been obtained:

(1) From 2010 to 2019, the overall poverty reduction efficiency of poverty counties exhibited an increasing trend with significant spatial heterogeneity. The poverty reduction efficiency of state poverty counties had increased most rapidly in the western region. TE and SE slightly increased by 0.5% and 2.2%, respectively. Technical efficiency (TE) and scale efficiency (SE) in the eastern and central regions remained largely unchanged, while the technical efficiency in the western region contributed significantly to the overall poverty reduction efficiency.

(2) From 2010 to 2019, the values of ecosystem services in state poverty counties have shown a downward annual trend. In 2010–2014 and 2010–2019, ecosystem services intensity declined by 1.51% and 0.80%, mainly in Tibet, Chengdu, Chongqing, the middle reaches of the Yangtze River, and the southwestern region.

(3) Different dimensions of ecosystem services have different effects on poverty reduction efficiency, which vary regionally. Ecosystem services have a significant positive impact on poverty reduction efficiency. Food production and carbon storage have significant positive effects on poverty reduction performance, while habitat quality has a significant negative impact. Aside from natural factors, tourism income and education expenditure have a significant positive relationship with poverty reduction efficiency, while agricultural output value and financial loans have a significant negative effect. Based on the empirical results, the framework of the ecology–economy–society mechanism of the poverty reduction efficiency in state poverty counties developed in this study can be used to support high-quality development and rural revitalization.

## Figures and Tables

**Table 1:** DEA model-specific indicators of poverty reduction efficiency in state poverty counties.

	Indicators	Indicator Symbol	Explanation
Input indicators	financial fund investment	financial	per capita expenditure of fiscal funds (yuan/person)
	the amount of employment	employment	the amount of employment in the secondary and tertiary industries (10,000 per person)
	construction land	urbanland	area of regional construction land (m^2^)
	research and development expenditure	rd	internal expenditure for research and experimental development (CNY 10,000)
Output indicators	net income per capita	perincome	per capita net income of the region (yuan/person)
	non-poverty incidence	poverty	single-poverty incidence

Note: ① The actual expenditure of the survey unit for internal research and development activities (basic research, applied research, and experimental development) includes direct expenditures for research and development project activities and those indirectly used for research and development activities, such as management fees, service fees, research and development-related capital construction expenditures, and outsourcing processing fees. ② Poverty incidence rate refers to the ratio of the poor population (that is, the population below the poverty line) to the total population, reflecting the scope of poverty in the region.

**Table 2:** Selection and description of variables.

Variable Type	Variable Symbol	Explanation
Comprehensive efficiency of poverty reduction	PRE	calculated from Section 3.2.1 of this paper
Technical efficiency	TE	
Scale efficiency	SE	
Ecosystem services	ES	calculated from Section 3.2.2 of this paper
Habitat quality	HQ	
Food production	FP	
Carbon storage	CS	
Precipitation	preci	regional annual rainfall (mm)
Temperature	temp	regional annual average temperature (degrees Celsius)
Vegetation index	ndvi	regional vegetation index
Agricultural output value	agri	regional agricultural output value (CNY 10,000)
Tourism income	tourism	regional tourism revenue (CNY 100,000,000)
Education expenditure	edu	regional education expenditure (CNY 10,000)
Financial loans	debt	regional financial loan line (CNY 10,000)

**Table 3:** National PRE and ecological service value: benchmark regression.

	PRE		TE		SE	
	(1)	(2)	(3)	(4)	(5)	(6)
ES	0.238 ***	0.274 ***	0.055 **	0.063 **	0.205 ***	0.247 ***
	(10.231)	(12.752)	(2.183)	(3.552)	(6.850)	(9.719)
tourism		0.004 ***		0.012 ***		−0.005 ***
		(3.259)		(9.884)		(−3.915)
edu		0.009 ***		0.006 ***		0.013 ***
		(7.085)		(4.602)		(9.043)
debt		−0.026 ***		0.031 ***		−0.055 ***
		(−11.431)		(13.124)		(−20.026)
preci		−0.020 ***		−0.004		−0.017 ***
		(−3.765)		(−0.726)		(−2.690)
temp		0.003		−0.016 ***		0.016 ***
		(1.127)		(−5.786)		(4.946)
ndvi		0.009		0.141 ***		−0.108 ***
		(0.384)		(5.534)		(−3.661)
agri		−0.011 ***		−0.009 ***		0.020 ***
		(−6.423)		(−5.106)		(9.905)
cons	0.801 ***	1.210 ***	1.002 ***	0.666 ***	0.801 ***	1.512 ***
	(66.208)	(23.677)	(76.938)	(12.486)	(51.460)	(24.471)
Year and region effect	control	control	control	control	control	control
N	2487	2478	2487	2478	2487	2478

Note: The statistical test values are in parentheses. **, *** indicate significance at the 5%, 1% levels, respectively.

**Table 4:** Robustness analysis: replacing the dependent variable.

	PRE_dum	TE_dum	SE_dum
	(1)	(2)	(3)
ES	0.919 ***	1.053 ***	1.292 ***
	(4.605)	(5.639)	(6.466)
tourism	0.011 ***	0.013 ***	0.001
	(2.687)	(2.585)	(0.285)
edu	0.001	0.040 ***	0.039 ***
	(0.274)	(8.014)	(8.881)
debt	−0.013 *	0.156 ***	−0.151 ***
	(−1.667)	(13.736)	(−12.472)
preci	−0.041 **	−0.046 **	−0.068 ***
	(−2.056)	(−2.055)	(−3.227)
temp	0.010	−0.063 ***	0.031 **
	(0.835)	(−4.663)	(2.440)
ndvi	0.240 **	0.504 ***	−0.038
	(2.433)	(4.815)	(−0.368)
agri	−0.002	−0.056 ***	0.049 ***
	(−0.326)	(−7.289)	(6.726)
Year and region effect	control	control	control
N	2478	2478	2478

Note: ① The regression results in the table are based on the logit model. The values indicate the marginal effect. ② *, **, and *** indicate significance at the 10%, 5%, and 1% levels, respectively. The numbers in parentheses are the z-statistic values.

**Table 5:** National PRE and ecological service value: sub-dimensions analysis.

	Explained Variable: PRE		
	(1)	(2)	(3)
HQ	−0.128 ***		
	(−7.009)		
FP		0.111 ***	
		(12.842)	
CS			0.092 ***
			(5.574)
cons	1.179 ***	1.170 ***	1.218 ***
	(22.924)	(23.065)	(23.600)
Control variable	control	control	control
Year and region effect	control	control	control
N	2478	2478	2478

Note: The statistical test values are in parentheses. *** indicates significance at the 1% levels.

**Table 6:** The impact of ES on the PRE in state poverty counties: different regions analysis.

	Explained Variable: PRE		
	Eastern Region	Central Region	Western Region
	(1)	(2)	(3)
ES	0.4527 **	0.3635 ***	0.2342 ***
	(2.36)	(3.49)	(5.05)
cons	1.5758 ***	0.8697 ***	1.3078 ***
	(4.95)	(7.34)	(22.54)
Control variable	control	control	control
Year and region effect	control	control	control
N	150	633	1695

Note: The statistical test values are in parentheses. **, *** are 5%, 1% significance levels, respectively.

## Data Availability

The data presented in this study are available on request from the corresponding author. The data are not publicly available due to the data also forms part of an ongoing study.

## Appendix A

**Table A1:** Analysis of the stationarity of variables in the panel model.

Variables	Homogeneous Panel LLC		Homogeneous Panel LLC		Conclusion
	T-Value	Significance	W-Value	Significance	
PRE	−14.114	0.000	−4.305	0.000	stationary
TE	−11.898	0.000	−4.017	0.000	stationary
SE	−14.126	0.000	−3.517	0.000	stationary
ES	−8.518	0.000	−5.785	0.000	stationary
HQ	−7.134	0.000	−4.003	0.000	stationary
FP	−12.101	0.000	−3.433	0.000	stationary
CS	−4.715	0.000	−4.621	0.000	stationary
preci	−3.372	0.000	−3.352	0.000	stationary
temp	0.265	0.000	−4.533	0.000	stationary
ndvi	−14.114	0.000	−4.305	0.000	stationary
agri	−11.898	0.000	−4.017	0.000	stationary
touism	−14.126	0.000	−3.517	0.000	stationary
edu	−8.518	0.000	−5.785	0.000	stationary
debt	−7.134	0.000	−4.003	0.000	stationary

**Table A2:** Descriptive statistics of variables in the panel model.

Variables	Mean	Standard Deviation	Minimum	Maximum
PRE	0.9220	0.1157	0.5630	1.4240
TE	1.0303	0.1222	0.6960	1.4020
SE	0.9060	0.1473	0.5850	1.2020
ES	0.5101	0.0976	0.0372	0.7172
HQ	0.6910	0.1596	0.0788	0.9806
FP	0.3632	0.2739	0	1
CS	0.4243	0.1378	0	1
preci	8.9753	0.6027	5.5114	10.2416
temp	4.4286	0.9884	−4.1412	5.5489
ndvi	0.7093	0.1873	0.0521	0.8950
agri	11.9613	1.5203	7.6059	13.0878
touism	5.1462	1.9437	0.8963	13.2631
edu	11.3502	1.9221	4.1146	18.3481
debt	13.1973	1.1126	10.2601	16.3805

**Table A3:** Coefficients in the panel model.

Variables	PRE	ES	preci	temp	ndvi	agri	tourism	edu	debt
PRE	1								
ES	0.2010 ***	1							
preci	0.0909 ***	0.1205 ***	1						
temp	0.1160 ***	0.2822 ***	0.1581 ***	1					
ndvi	0.1640 ***	0.2530 ***	0.2825 ***	0.1037 ***	1				
agri	0.0456 ***	0.1166 ***	0.0375 **	0.0830 ***	0.0969 ***	1			
touism	0.0580 ***	0.0267 *	−0.0437 *	0.1168 **	0.0526 ***	0.0114 *	1		
edu	−0.0568 **	0.2924 ***	0.042 ***	0.1434 ***	0.2370 ***	0.0630 ***	0.0635 ***	1	
debt	−0.1759 ***	0.0207 *	−0.0211 *	−0.0582 ***	0.0246 *	0.1691 ***	0.0467 ***	0.0347 ***	1

Note: *, **, and *** indicate 10%, 5%, and 1% levels of significance, respectively.

## References

[1] Liu Y., Wang Y. (2019). Rural land engineering and poverty alleviation: Lessons from typical regions in China. J. Geogr. Sci..

[2] Wan G., Hu X., Liu W. (2021). China’s poverty reduction miracle and relative poverty: Focusing on the roles of growth and inequality. China Econ. Rev..

[3] Shuai J., Liu J., Cheng J., Cheng X. (2021). Interaction between ecosystem services and rural poverty reduction: Evidence from China. Environ. Sci. Policy.

[4] Wang H., Wen T., Han J. (2020). Can Rural Households, Loan Effectively Improve the Quality of Poverty Alleviation in Areas of Extreme Poverty?. Chin. Rural. Econ..

[5] Kieslich M., Salles J. (2021). Implementation context and science-policy interfaces: Implications for the economic valuation of ecosystem services. Ecol. Econom..

[6] Fisher J.A., Patenaude G., Giri K., Lewis K., Meir P., Pinho P., Rounsevell M.D.A., Williams M. (2014). Understanding the relationships between ecosystem services and poverty alleviation: A conceptual framework. Ecosyst. Serv..

[7] Chen Y., Xia Q., Wang X. (2021). Consumption and Income Poverty in Rural China: 1995–2018. China World Econ..

[8] Alkire S., Foster J. (2011). Counting and multidimensional poverty measurement. J. Public Econ..

[9] Gallardo M. (2013). Using the downside mean-semideviation for measuring vulnerability to poverty. Econ. Lett..

[10] Si L., Wang C. (2020). Measurement of Regional Poverty Alleviation Quality and its Spatiotemporal Evolution: A Study Based on Night Light Data of Poor Counties. J. Macro-Qual. Res..

[11] Wang Z., Li J., Liu J., Shuai C. (2020). Is the photovoltaic poverty alleviation project the best way for the poor to escape poverty?—A DEA and GRA Analysis of Different Projects in Rural China. Energy Policy.

[12] Gu N., Liu Y. (2021). Does Industrial Poverty Alleviation Reduce Poverty Vulnerability of Poor Households?. J. Agrotech. Econ..

[13] Abosedra S., Shahbaz M., Nawaz K. (2015). Modeling Causality between Financial Deepening and Poverty Reduction in Egypt. Soc. Indic. Res..

[14] Zhao L. (2020). Tourism, Institutions, and Poverty Alleviation: Empirical Evidence from China. J. Travel. Res..

[15] Xie Y., Xie E. (2020). Comparing Income Poverty with Multidimensional Well-being Based on the “Conversion Efficiency”. Soc. Indic. Res..

[16] Baloch M.A.B., Danish, Khan S.U.-D.K., Ulucak Z.Ş., Ahmad A. (2020). Analyzing the relationship between poverty, income inequality, and CO_2_ emission in Sub-Saharan African countries. Sci. Total Environ..

[17] Jiang A., Chen C., Ao Y., Zhou W. (2021). Measuring the Inclusive growth of rural areas in China. Appl. Econ..

[18] Tavares F.F., Betti G. (2021). The Pandemic of Poverty, Vulnerability, and COVID-19: Evidence from a Fuzzy Multidimensional Analysis of Deprivations in Brazil. World Dev..

[19] Yao Y., Sun J., Tian Y., Zheng C., Liu J. (2020). Alleviating water scarcity and poverty in drylands through telecouplings: Vegetable trade and tourism in northwest China. Sci. Total Environ..

[20] Koomson I., Danquah M. (2021). Financial inclusion and energy poverty: Empirical evidence from Ghana. Energy Econ..

[21] Wang F., Zhen H., Zhang W., Wang H., Peng W.-J. (2021). Regional differences and their driving mechanism of relationships between rural household livelihood and ecosystem services: A case study in upstream watershed of Miyun Reservoir, China. J. Appl. Ecol..

[22] Zhao S., Wu X., Zhou J., Pereira P. (2021). Spatiotemporal tradeoffs and synergies in vegetation vitality and poverty transition in rocky desertification area. Sci. Total Environ..

[23] Caldés N., Coady D., Maluccio J.A. (2006). The Cost of Poverty Alleviation Transfer Programs: A Comparative Analysis of Three Programs in Latin America. World Dev..

[24] Li M. (2010). Decomposing the change of CO_2_ emissions in China: A distance function approach. Ecol. Econom..

[25] Meng S., Zhou W., Chen J., Zhang C. (2018). A synthesized data envelopment analysis model and its application in resource efficiency evaluation and dynamic trend analysis. Energy Environ..

[26] Ouyang X., Tang L., Wei X., Li Y. (2021). Spatial interaction between urbanization and ecosystem services in Chinese urban agglomerations. Land Use Policy.

[27] Di Febbraro M., Sallustio L., Vizzarri M., De Rosa D., De Lisio L., Loy A., Eichelberger B.A., Marchetti M. (2018). Expert-based and correlative models to map habitat quality: Which gives better support to conservation planning?. Glob. Ecol. Conserv..

[28] Zhang D., Huang Q., He C., Wu J. (2017). Impacts of urban expansion on ecosystem services in the Beijing-Tianjin-Hebei urban agglomeration, China: A scenario analysis based on the Shared Socioeconomic Pathways. Resour. Conserv. Recycl..

[29] Sharp R., Tallis H.T., Ricketts T., Guerry A.D., Wood S.A., ChaplinKramer R., Nelson E., Ennaanay E., Wolny S., Olwero N. (2014). InVEST User’s Guide.

[30] Peng B., Yu J., Zhu Y. (2021). A heteroskedasticity robust test for cross-sectional correlation in a fixed effects panel data model. Econ. Lett..

[31] Majeed A., Wang L., Zhang X., Kirikkaleli D. (2021). Modeling the dynamic links among natural resources, economic globalization, disaggregated energy consumption, and environmental quality: Fresh evidence from GCC economies. Resour. Policy.

[32] Li Y., Zhang Q., Wang G., Liu X., Mclellan B. (2019). Promotion policies for third party financing in Photovoltaic Poverty Alleviation projects considering social reputation. J. Clean. Prod..

[33] Cheng X., Chen J., Jiang S., Dai Y., Shuai C., Li W., Liu Y., Wang C., Zhou M., Zou L. (2021). The impact of rural land consolidation on household poverty alleviation: The moderating effects of human capital endowment. Land Use Policy.

[34] Pan Y., Wu J., Zhang Y., Zhang X., Yu C. (2021). Simultaneous enhancement of ecosystem services and poverty reduction through adjustments to subsidy policies relating to grassland use in Tibet. China. Ecosyst. Serv..

[35] Haider L.J., Boonstra W.J., Peterson G.D., Schlüter M. (2018). Traps and Sustainable Development in Rural Areas: A Review. World. Dev..

[36] Suich H., Howe C., Mace G. (2015). Ecosystem services and poverty alleviation: A review of the empirical links. Ecosyst. Serv..

[37] Bathla S., Joshi P.K., Kumar A. (2019). Targeting Agricultural Investments and Input Subsidies in Low-Income Lagging Regions of India. Eur. J. Dev. Res..

[38] Liu F., Li L., Zhang Y., Ngo Q.T., Iqbal W. (2021). Role of education in poverty reduction: Macroeconomic and social determinants form developing economies. Environ. Sci. Pollut. Res..

[39] Nizam R., Karim Z.A., Rahman A.A., Sarmidi T. (2020). Financial inclusiveness and economic growth: New evidence using a threshold regression analysis. Econ. Res. Ekon. Istraživanja.

[40] Neaime S., Gaysset I. (2018). Financial inclusion and stability in MENA: Evidence from poverty and inequality. Financ. Res. Lett..

[41] Liu M., Feng X., Wang S. (2021). Does poverty-alleviation-based industry development improve farmers’ livelihood capital?. J. Integr. Agric..

